# Nonlinear refinement of functional brain connectivity in golf players of different skill levels

**DOI:** 10.1038/s41598-022-06161-3

**Published:** 2022-02-11

**Authors:** Tai-Ting Chen, Kuo-Pin Wang, Chung-Ju Huang, Tsung-Min Hung

**Affiliations:** 1 Department of Physical Education and Sport Sciences, National Taiwan Normal University, No. 162, Section 1, Heping East Road, Da-an District, Taipei, 106 Taiwan, ROC; 2 Center for Cognitive Interaction Technology, Bielefeld University, Inspiration 1, 33619 Bielefeld, Germany; 3 Neurocognition and Action - Biomechanics Research Group, Faculty of Psychology and Sports Science, Bielefeld University, Universitätsstraße 25, 33615 Bielefeld, Germany; 4 Graduate Institute of Sport Pedagogy, University of Taipei, No. 101, Section 2, Zhongcheng Road, Shilin District, Taipei, 111 Taiwan, ROC; 5 Institute for Research Excellence in Learning Science, National Taiwan Normal University, No. 162, Section 1, Heping East Road, Da-an District, Taipei, 106 Taiwan, ROC

**Keywords:** Neuroscience, Psychology

## Abstract

Different functional connectivities in the brain, specifically in the frontoparietal and motor cortex–sensorimotor circuits, have been associated with superior performance in athletes. However, previous electroencephalogram (EEG) studies have only focused on the frontoparietal circuit and have not provided a comprehensive understanding of the cognitive–motor processes underlying superior performance. We used EEG coherence analysis to examine the motor cortex–sensorimotor circuit in golfers of different skill levels. Twenty experts, 18 amateurs, and 21 novices performed 60 putts at individual putting distances (40–60% success rate). The imaginary inter-site phase coherence (imISPC) was used to compute 8–13 Hz coherence that can be used to distinguish expert-novice and expert-amateur differences during motor preparation. We assessed the 8–13 Hz imISPC between the Cz and F3, F4, C3, C4, T3, T4, P3, P4, O1, and O2 regions. (1) Amateurs had lower 8–13 Hz imISPC in the central regions (Cz–C3 and C4) than novices and experts, but experts had lower 8–13 Hz imISPC than novices. (2) Skilled golfers (experts and amateurs) had lower 8–13 Hz imISPC in the central–parietal regions (Cz–P3 and P4) than novices. (3) Experts had lower 8–13 Hz imISPC in the central–left temporal regions (Cz–T7) than amateurs and novices. Our study revealed that refinement of the motor cortex–sensorimotor circuit follows a U-shaped coherence pattern based on the stage of learning. The early learning stage (i.e., novice to amateur) is characterized by lower connectivity between the regions associated with motor control and visuospatial processes, whereas the late learning stage (i.e., amateur to expert) is characterized by lower connectivity in the regions associated with verbal-analytic and motor control processes.

## Introduction

Compared with novice athletes, experts exhibit superior integration of perceptual (e.g., quiet eye)^1^, cognitive (e.g., sense of distance)^2^, and motor skills (e.g., motor control)^3^. This superior ability has been associated with the focused and efficient organization of task-related neural networks^4^. It is clear that long-term training helps attain a high level of expertise that is associated with the refinement of visuomotor integration processes^4,5^. A valuable model for the interpretation of these processes has been proposed by Hikosaka et al.^6^, in which two loop circuits, frontoparietal and motor cortex–sensorimotor, function in parallel. The frontoparietal circuit reflects visuospatial coordinates and the motor cortex–sensorimotor circuit processes motor coordination^6^. These two circuits are known to contribute to the improvement of performance. That is, the refinement of visuospatial and motor coordination processing is essential to achieve highly skilled performance levels^7^. As the frontoparietal circuit has been associated with visuospatial processes and the motor cortex–sensorimotor motor circuit reflects motor processes, examining these two circuits could enable differentiation between skill levels. Therefore, it is critical to uncover the role of both circuits in the development of skilled performance to understand the contribution of cognitive–motor processes in motor learning and performance. Previous functional magnetic resonance imaging (fMRI) studies have used functional connectivity analysis to identify the two parallel loop circuits underlying skilled performance. Kincses et al.^8^ and Hikosaka et al.^6^ revealed that both circuits undergo changes in connectivity during motor learning^9^. However, fMRI is limited by low temporal resolution, making it difficult to pinpoint the precise moments of dynamic neural activity. The inability of fMRI to capture the electrical signals that define neuronal communication render it a poor technique by which to examine the highly dynamic neural activities that occur during the essential preparatory stages (e.g., 2 s before action) of skilled performances^10^.

Electroencephalogram (EEG) coherence compensates for the limitations of fMRI and can record the highly dynamic neuromotor processes that occur during and after the motor preparation stage^11–13^. Indeed, EEG coherence has previously been used to elucidate the functional connectivity of visuomotor integration processes during the essential preparatory stage in highly skilled performers^10,14^. When analyzing EEG coherence data, higher coherence is thought to indicate stronger cortico–cortical communication, whereas lower coherence is thought to indicate cortical autonomy. Past EEG studies that adopted the expert-novice or expert-amateur paradigms have also examined the frontoparietal circuit. For example, when compared to novices, experts show reduced communication between the motor planning (frontal regions: F3, F4) and visuospatial attention regions (parietal regions: P3, P4), as reflected by lower low-alpha (8–10 Hz) and high-alpha (11–13 Hz) coherence^15^. However, no significant differences have been found between the frontoparietal circuits of expert and amateur shooters^16^, implying that skilled performers refine their visuospatial coordinates (reflected as decreased neuromotor noise) by decreasing communication between the frontal and parietal regions to attend to the motor, somatosensory, and visual demands of the task. These findings support the model proposed by Hikosaka et al.^6^ and draw attention to the frontoparietal circuit in the expert-novice or expert-amateur paradigms. However, focusing only on the frontoparietal circuit cannot provide a comprehensive picture of the cognitive–motor processes underlying superior performance.

Given that the motor cortex–sensorimotor circuit is one of the key players in superior performance, the communication that occurs within this circuit during the skilled performance of motor activities should be examined^7^. The mu rhythm (8–13 Hz), located in the central region (e.g., C3, Cz, C4), is associated with sensorimotor processes^17^, and mainly regulates motor coordination activities such as voluntary motor control, direction, and force. Accordingly, 8–13 Hz can be a determinant of skilled motor performance in precision sports activities, such as putting in golf^10,18–20^. Only two EEG studies have investigated the motor cortex–sensorimotor circuit in performers of different skill levels. Deeny et al.^16^ compared expert marksmen with skilled shooters and found that expert marksmen had lower coherence between the central and left temporal regions. Del Percio et al.^14^ observed that event-related 8–12 Hz coherence in the central–parietal region was not significantly different between elite air pistol shooters and novices. Unfortunately, even though motor coordination processing plays an essential role in superior performance, these studies could not provide detailed information on cortico–cortical communication in the motor cortex–sensorimotor circuit^7^. Therefore, further investigation of the motor cortex–sensorimotor circuit is required to reveal the key cognitive mechanisms underlying superior athletic performance.

Currently, EEG coherence studies that perform expert-novice or expert-amateur comparisons have two major limitations. First, these studies utilize spectral coherence, which cannot eliminate volume conduction and can be affected by strong increases or decreases in EEG power^21^. However, the imaginary inter-site phase coherence (imISPC)^21,22^, which is not directly linked to power, can be used to address this potential confounding factor. Second, EEG coherence studies based on expert-novice or expert-amateur paradigms do not fully account for the cognitive–motor processes underlying skilled performance that arise from the dynamic and nonlinear refinement of brain activity across different stages of learning^7,23^. Therefore, it is possible that the activation pattern takes an inverted U-shape, depending on the stage of learning^10,23,24^. By using the expert-amateur-novice design and analyzing 8–13 Hz imISPC in the motor cortex–sensorimotor circuit, one can extend the previous findings and specify the cognitive–motor processes underlying superior performance.

In this study, we examined the motor cortex–sensorimotor circuit as motor coordination processing is thought to be an essential cognitive–motor process that facilitates the attainment of highly skilled performance levels^7^. We used 8–13 Hz imISPC to examine the dynamic neural activity during the essential preparatory stage (e.g., 2 s before action) across golfers of different skill levels. This provided a comprehensive picture of the cognitive–motor processes underlying superior performance. Furthermore, the level of task difficulty employed in this study (i.e., only half of the putts were successful), induced the integration of sensorimotor and task-relative attentional processes during motor preparation^10,20^. Previous fMRI studies have shown the dynamic reorganization of the motor cortex–sensorimotor circuit during different stages of motor skill learning^6–8,25^. The early motor skill learning stage (e.g., corresponding to the cognitive and associative stages) is characterized by increased functional connectivity in the motor cortex–sensorimotor circuit. The late learning stage (e.g., corresponding to the associative and automatic stages), however, is associated with decreased connectivity in the motor cortex–sensorimotor circuit. Therefore, we expected amateurs to have higher 8–13 Hz imISPC in the central regions (C3, Cz, C4) than novices and experts; we also anticipated that experts would have lower 8–13 Hz imISPC in the central regions than novices during motor preparation. Given that other task-related regions (frontal, parietal, occipital, and temporal) are critical for high performance levels in precision sports such as golf putting^10,19^, we also examined 8–13 Hz imISPC simultaneously in multiple regions of interest.

## Results

### EEG

**Figure 1:**
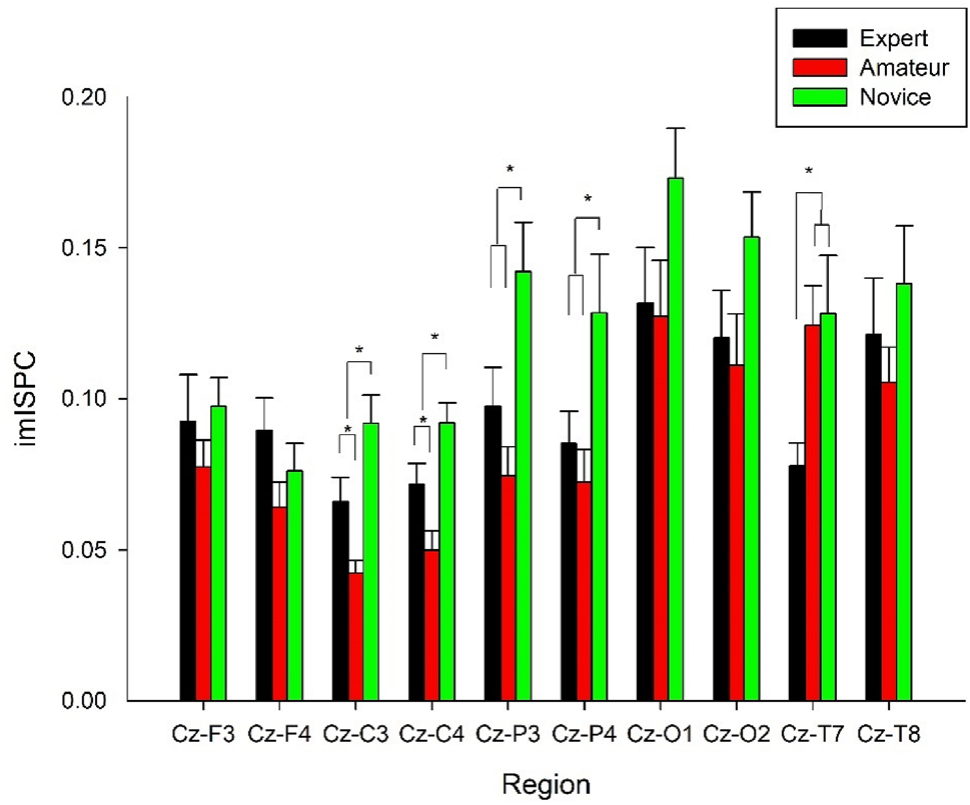
Mean values (± standard error [SE]) of 8–13 Hz imaginary inter-site phase coherence (imISPC) in experts (black bar), amateurs (red bar), and novices (green bar) for the electrode pairings of interest. *Significant difference, *p* < 0.05, false discovery rate (FDR) corrected.

A 3 group × 2 hemisphere × 5 region 3-way multivariate analysis of covariance (MANCOVA) of 4–7 Hz, 8–13 Hz, and 14–20 Hz ImISPC revealed a significant group × hemisphere × region interaction: *F*(24, 88) = 1.643, *p* = 0.050, Wilks’ lambda = 0.477, *η*_*p*_^2^ = 0.309, power = 0.949. However, only the univariate analysis of 8–13 Hz showed a significant group × hemisphere × region interaction: *F*(8, 220) = 3.419, *p* = 0.001, *η*_*p*_^2^ = 0.111, power = 0.976). A simple effect analysis demonstrated a significant interaction of group × region in the left hemisphere (*F*(8, 220) = 2.214, *p* = 0.027, *η*_*p*_^2^ = 0.075, power = 0.858). A simple main effect analysis revealed a significant group effect at Cz–C3 (*F*(2, 56) = 10.416, *p* < 0.001), at Cz–P3 (*F*(2, 56) = 6.387, *p* = 0.003), and at Cz–T7 (*F*(2, 56) = 3.848, *p* = 0.027). As can be seen in Fig. 1, post hoc analysis showed that (1) amateurs had lower 8–13 Hz coherence at Cz–C3 than novices (*p* = 0.015, *d* = 1.53) and experts (*p* = 0.049, *d* = 0.84), and the experts had lower coherence than novices (*p* = 0.045, *d* = 0.66); (2) novices had higher 8–13 Hz coherence at Cz–P3 than experts (*p* = 0.045, *d* = 0.66) and amateurs (*p* < 0.001, *d* = 1.12), but there were no significant differences between the experts and amateurs (*p* = 0.253, *d* = 0.45); and (3) experts had lower 8–13 Hz coherence at Cz–T7 than amateurs (*p* = 0.048, *d* = 1.01) and novices (*p* = 0.045, *d* = 0.75), but there were no significant differences between amateurs and novices (*p* = 0.848, *d* = 0.05). A simple effect analysis demonstrated a significant interactive effect of group × region in the right hemisphere (*F*(8, 220) = 2.054, *p* = 0.042, *η*_*p*_^2^ = 0.070, power = 0.826). A simple main effect analysis revealed a significant group effect at Cz–C4 (*F*(2, 56) = 9.998, *p* < 0.001) and at Cz–P4 (*F*(2, 56) = 4.038, *p* = 0.023). Post hoc analysis showed that (4) amateurs had lower 8–13 Hz coherence at Cz–C4 than novices (*p* = 0.005, *d* = 1.47) and experts (*p* = 0.048, *d* = 0.74), and the experts also had lower 8–13 Hz coherence than novices (*p* = 0.046, *d* = 0.67); and (5) novices had higher 8–13 Hz coherence at Cz–P4 than experts (*p* = 0.048, *d* = 0.60) and amateurs (*p* = 0.037, *d* = 0.78), but there were no significant differences between experts and amateurs (*p* = 0.587, *d* = 0.27).

#### Task specificity

A 3 (group: experts, amateurs, novices) × 2 (hemisphere: left, right) × 5 (region: frontal, central, parietal, occipital, temporal) ANOVA indicated no significant group × hemisphere × region interaction effect (*p* = 0.542). Furthermore, neither the group × hemisphere (*p* = 0.428) nor the group × region interaction effect (*p* = 0.179) was statistically significant.

## Discussion

This study used 8–13 Hz imISPC to characterize the cortico–cortical connections in the motor cortex–sensorimotor circuit between central and other regions in three different levels of golf players. Through this analysis, we were able to gain critical information on the visuomotor integration required for superior performance. We found that (1) amateurs had lower 8–13 Hz imISPC in the central regions (Cz–C3 and C4) than novices and experts, but experts had lower 8–13 Hz imISPC than novices; (2) skilled golfers (experts and amateurs) had lower 8–13 Hz imISPC in the central–parietal regions (Cz–P3 and P4) than novices; and (3) experts had lower 8–13 Hz imISPC in the central–left temporal regions (Cz–T7) than amateurs and novices. This study extends previous research as, until now, few studies have examined the motor cortex–sensorimotor circuit in athletes of different skill levels.

One of our major findings was that amateurs have lower 8–13 Hz imISPC in the central regions (Cz–C3 and C4) than novices and experts, but that experts have lower 8–13 Hz coherence than novices. This implied that the refinement of the motor cortex–sensorimotor circuit is likely to follow a U-shaped coherence curve, depending on the stage of learning. The Cz–C3 and C4 regions are associated with motor programming events such as movement force and movement direction^19,26^. Increased communication between these regions may indicate that movement coordination is being augmented during motor preparation^7^. However, our finding conflicts with those of earlier fMRI studies, which have reported that the early learning stage is associated with increased integration in the motor cortex–sensorimotor circuit and that the late learning stage is associated with decreased integration in this circuit. This discrepancy between our study and previous studies may reflect differences between the short- and long-term effects of training. For example, previous fMRI studies have tracked functional connectivity over periods of 1 day to 4 weeks of training in motor tasks^8,9,25,27^. However, in our study, both the amateur and expert golfers had 2–8 years of experience, which is deemed to represent long-term training. The results of earlier fMRI studies may therefore be equivalent to the comparison of novices to amateurs in our study. Thus, our study provides a more comprehensive understanding of the motor cortex–sensorimotor circuit at all stages of learning (cognitive, associative, and autonomous). From the cognitive perspective, and based on the three stages of motor learning^28^ and the three stages of knowledge^29^, we infer that a novice with no experience in golf putting performs inefficient processing during a golf putting task. During the corresponding cognitive stage (comparable to the declarative knowledge stage), a novice would mainly process the rules of golf putting^29^ and consequently would have relatively unstable neural processes^4^. This inference implies that novices engage their movement programming processes to a larger extent than do skilled golfers at both the amateur and expert levels. Among skilled performers, amateurs are in the associative stage, in which they attempt to translate declarative knowledge into procedural knowledge (i.e., from “what to do” to “how to do it”)^30^. Essentially, as the declarative knowledge of their movements decreases^29^, amateurs attempt to perform basic golf putting skills efficiently and with technical accuracy, thereby reducing the coordination of movement programming processes^10^. Experts attempt to withdraw gradually from cognitive analysis of procedural knowledge and progress to mostly automatic processes^28^. It is therefore reasonable to assume that an expert has established a well-developed internal model through which a strong memory representation is formed by repeatedly negotiating the demands of the task^31,32^. This model allows experts to perform skills using strategic knowledge (i.e., the ability to recognize and respond optimally in various conditions)^29^, resulting in the specific and functional coordination of movement programming processes during motor preparation^10^. Our findings extend those of previous fMRI studies by adopting an expert–amateur–novice design and providing evidence of dynamic reorganization of the motor cortex–sensorimotor circuit in association with superior skilled performance^6,7^.

Interestingly, we observed that skilled performers (experts and amateurs) had lower 8–13 Hz imISPC between the central and parietal areas (P3 and P4) than novices during putting preparation, but there were no significant differences in coherence between skilled golfers (experts and amateurs). This indicates that, compared to novices, skilled performers show a lower degree of cortico–cortical communication, particularly between the sensorimotor and visuospatial information regions. This further implies a lower degree of involvement of visuospatial processes in motor control processes. Our study complements those conducted by Del Percio et al.^14^. Del Percio et al.^14^ observed that event-related 8–12 Hz coherence in the central–parietal regions was not significantly different between skilled air pistol shooters and novices. These results when combined with ours suggest that skilled performers have a lower degree of connectivity between the visuospatial and motor control processes during motor preparation. Skilled performers, therefore, appear to demonstrate neural efficiency when connecting visuospatial with motor control processes owing to their well-organized mental representations of putting skills in their long-term memories^32^. However, we also observed that novices focused on the essential tips for putting. This echoes the psychomotor efficiency hypothesis, which assumes that superior performance is associated with the selective downregulation of certain processes during motor preparation^24^.

We observed that expert golfers have lower 8–13 Hz imISPC between the central and left temporal regions (T7), although there were no significant differences between the amateurs and novices. This result extends those of previous studies that examined the frontal and left temporal regions in the expert-novice^15^ and expert-amateur paradigms^10^. In addition, this result confirms the observations made by Deeny et al.^16^, who reported that expert marksmen had lower coherence between the central and left temporal regions than amateur shooters. The left temporal region is associated with verbal-analytical processes^15,16,33^ and is involved in stimulus feature detection^34^. Skilled performers show decreased communication between the left temporal and central regions, implying that experts reduce the engagement of verbal-analytical processes with motor control processes either by reducing the overall engagement with verbal-analytic processes or by explicitly monitoring the elements of performance^24^. Specifically, to maintain performance effectiveness for the level of task difficulty presented in this study (40–60% success rate), expert golfers need to carefully monitor and appropriately regulate their movements while being aware of their current experiences and not influencing the course of action. Therefore, it is not surprising that expert golfers showed lower connectivity between the verbal-analytic and motor control processes during motor preparation. These findings suggest that the decreased input from verbal-analytical processes during motor monitoring under challenging conditions may be an additional characteristic of high levels of psychomotor efficiency.

Overall, this study suggests that the motor cortex–sensorimotor circuit is an essential cognitive-motor process underlying skilled performance^8^. The refinement of the motor cortex–sensorimotor circuit appears to follow a U-shape coherence curve, depending on the stage of learning. Additionally, reduced connectivity between the motor control and visuospatial processes is a characteristic of the early learning stage (i.e., novice to amateur), whereas reduced connectivity between the verbal-analytic and motor control processes is associated with the late learning stage (i.e., amateur to expert). Our findings, obtained using 8–13 Hz imISPC, support a model in which the sensorimotor circuit is the major circuit that contributes to performance improvement^6,7^. We also extend the findings of previous studies, which have focused mainly on the frontoparietal circuit in performers with different skill levels^15,16^. In addition, these findings resonate with the psychomotor efficiency hypothesis, which proposes that the refinement of brain processes may be associated with the selective functional activation of task-relevant processes and the inhibition of task-irrelevant processes^24^. Different distances from the hole for each participant may be associated with different kinetics and kinematics of putting, possibly resulting in deleterious effects. We controlled for these differences in putting distance across the participants by using the distance as a covariate in our data analysis. Although we controlled for these confounding factors, the study had some limitations. Given the cross-sectional design of this study, our findings cannot define a causal relationship between cognitive–motor processes and superior performance. We recommend that future studies should manipulate these cognitive processes to establish causal relationships. A high-density EEG recording and source localization algorithm could also enable researchers to validate the involvement of these regions in the execution of skilled performances. Finally, we recommend that future studies include an amateur group to enable examination of neuromotor activity in players with different skill levels, because the refinement of cognitive-motor processes is likely to follow dynamic and nonlinear refinement mechanisms in the brain. For example, Chen et al. (2020) found an inverted U-shaped relationship between the activation of brain regions (right and left dMPC, left IPS/SPL, left pMTG/pSTS) and the years of experience in three groups of baseball batters with different skill levels (skilled, intermediate, novice). Similarly, Wang et al.^10^ showed that before putting, expert golfers demonstrated higher levels of attention than amateurs. The brain activation trends observed in these two studies are consistent with our findings and reflect the dynamic and nonlinear refinement of cognitive-motor processes. Therefore, future studies should include an amateur group to better elucidate the differences in cognitive development resulting from practice at different skill stages and the differences in information processing.

In conclusion, this study provides detailed information on the involvement of the motor cortex–sensorimotor circuit in skilled performance. Specifically, our findings support the assumption that dynamic and nonlinear refinement of the motor cortex–sensorimotor circuit characterizes the achievement of cognitive–motor processes during motor learning and superior performance^6,7^. Thus, the 8–13 Hz imISPC measure not only extends previous fMRI and EEG studies but also specifies the cognitive–motor processes underlying motor preparation.

## Methods

### Participants

The number of participants to be recruited was determined using power analysis software (G*Power 3.1) and repeated measures analysis of variance (ANOVA), with α = 0.05, power = 0.80, and effect size = 0.84^10^. Following from this, the minimum required sample size was calculated to be 33 participants. Given the potential power analysis biases that exist in neuroscience^35,36^, we recruited 59 participants for this study. The participants were classified into three groups: 20 experts (11 females, 9 males; mean age = 20.70 ± 2.05 years; mean golf experience = 8.15 ± 2.68 years), 18 amateurs (9 females, 9 males; mean age = 20.88 ± 1.81 years; mean golf experience = 2.77 ± 1.76 years), and 21 novices (11 females, 10 males; mean age = 22.47 ± 1.56 years). The mean handicaps for the expert and amateur golfers were 4.25 (standard deviation [SD] = 1.99) and 32.44 (SD = 6.21), respectively. The statistical analysis conducted by the United States Golf Association (USGA) posits that elite golfers have a handicap of 2.0–5.9^37^. Therefore, the expert golfers recruited for this study could be defined as elite golfers at a high competitive level^38,39^. All of the recruited participants met the following criteria: (1) right-hand dominant^40^; (2) no history of neurological disorders or related medication; and (3) no history of a high caffeine or alcohol consumption habit. All participants gave an informed written consent, and the study was approved by the Research Ethics Committee of National Taiwan Normal University. All methods were carried out in accordance with the relevant guidelines and regulations of Research Ethics Committee.

### Measures

#### Golf putting task

The golf putting task was as described in Wang et al.^10^ and consisted of the following components: (1) a regulation hole (diameter = 10.80 cm) on an artificial putting green (length = 6 m, width = 0.9 m); (2) standard-size golf balls (diameter = 4.27 cm); and (3) putting distance (calculated to allow a 40–60% success rate). The mean putting distances for the expert, amateur, and novice golfers were 422 cm (SD = 28), 345 cm (SD = 21.21), and 235 cm (SD = 39.95), respectively. The motor preparation period was defined as the time elapsed between the placement of the putter behind the ball and initiation of the backswing^41^. We used an infrared sensor to detected the backswing during each trial to obtain event-marker data. All of the participants used their own golf putters.

#### EEG recording

The EEG recording procedure was as described in Wang et al.^10^. Electrodes were positioned and signals were recorded in accordance with the standards of the international 10–20 system. We recorded EEGs continuously from 32 scalp locations (Fp1, Fp2, F7, F3, Fz, F4, F8, FT7, FC3, FCz, FC4, FT8, T7, C3, Cz, C4, T8, TP7, CP3, CPz, CP4, TP8, P7, P3, Pz, P4, P8, O1, Oz, O2), using the left and right mastoids (A1and A2) as a common average reference and FPz as a ground electrode^42^. We used a bipolar configuration to record vertical and horizontal electrooculograms (VEOG and HEOG, respectively). The EEG data were collected using NeuroScan NuAmps acquisition amplifiers (NeuroScan, Charlotte, NC, USA). Analog data were collected continuously at a sampling rate of 1000 Hz. The filter was set at 1–100 Hz, with the notch filter set at 60 Hz during the data collection. The impedance was maintained at < 5 KΩ at each electrode site.

### Procedures

The preliminary examination and testing were conducted on separate days so that the participants could familiarize themselves with the experiment. In the preliminary examination, the participants were (a) asked not to consume coffee or alcoholic beverages; (b) given an explanation of the purpose and the procedures of study; (c) asked to read and sign an informed consent form; (d) asked to put on a Lycra electrode cap; and (e) asked to practice 100 putts on the artificial green to get accustomed to the feel of the EEG cap during putting. Importantly, given that the novices had no prior putting experience, we showed them a teaching video to teach them how to putt. To ensure that all novices understood the tips for putting, they were asked to complete a golf questionnaire with 10 questions (100 marks) and were asked to attain 80/100 points before the practice sessions. If the individual did not get 80/100 points, the participant would be asked to watch the video again until the assessment score reaching 80 points. During the practice sessions (100 practice putts), the individual putting distance that would allow a 40–60% success rate was determined and used on the subsequent testing day. To determine the individual putting distance, we began with a putting distance of 200 cm and after 10 trial putts, performed an up or down distance adjustment of 20 cm if the average success rate of 10 putts was outside the 40–60% range. This procedure was repeated until the target success rate was achieved. On the testing day, all of the participants followed the same procedure as for the preliminary examination. To confirm that the individual putting distance would allow a 40–60% success rate, the participants performed 10 warm-up putts. After the appropriate putting distance was confirmed, they performed 6 blocks of putts, with each block containing 10 balls. The participants were allowed to rest for 2 min between blocks. For each trial, the backswing movement was detected using an infrared sensor to obtain event marker data.

### Data processing

**Figure 2:**
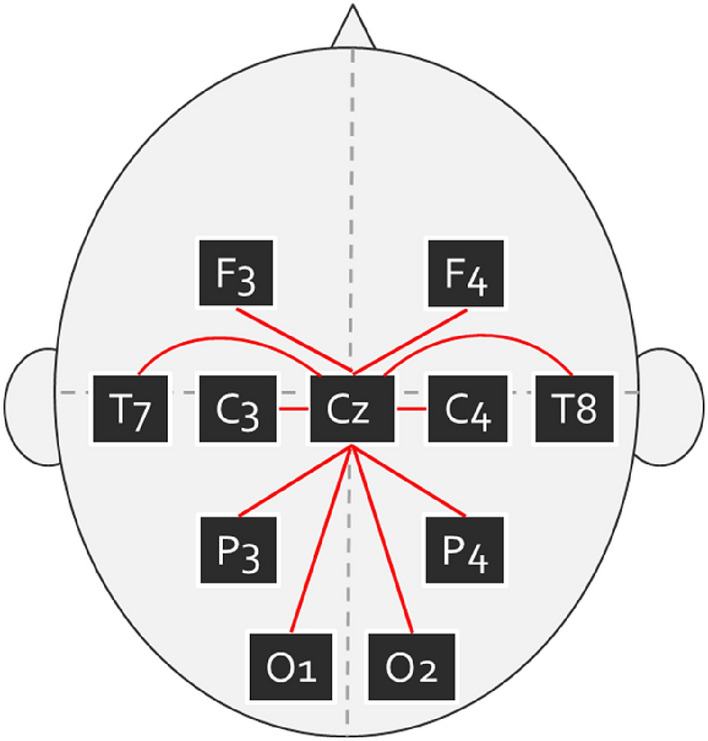
Left and right hemisphere electrode sites paired with Cz.

EEGLAB functions^43^ and custom scripts written in MATLAB (MathWorks, USA) were used to preprocess the offline EEG data. First, all of the EEG signals were re-referenced to the average of the mastoids (A1, A2). Second, band-pass filters ranging from 1 Hz (low-pass) to 30 Hz (high-pass) were used to perform basic finite infinite response (FIR) filtering. Third, we extracted data from the –2000 ms to 0 ms time period before defining epochs. Fourth, channels with bad signals were removed and interpolated before averaging; however, no bad channels were identified. Fifth, 64 trials were rejected (expert golfers = 25 ± 1.25 trials, amateur golfers = 11 ± 0.6 trials, and novices = 28 ± 1.3 trials) because they had epochs with amplitudes exceeding ± 100 µV, which may have been potential artifacts^44^. Finally, we utilized independent component analysis (ICA; Runica Infomax algorithm)^45^ to identify and remove potential artifacts (blinks, eye movements, other non-neural activity). After preprocessing the data, we used a complex Morlet wavelet convolution for time–frequency decomposition. The parameters of the complex Morlet wavelet (*CMW*) are $CMW={e}^{i2\pi ft}{e}^{{-\left(t\right)}^{2}/({2s}^{2})}$, where $e$ is the exponential; $i$ is the imaginary number; $f$ is the frequency, which ranged from 8 to 13 Hz in 6 logarithmically spaced steps; $t$ is time; and $s$ is the Gaussian width, calculated as $n/(2\uppi f)$, where *n* = 4 for the time–frequency precision trade-off^21^. The EEG connectivity was computed for each epoch using bespoke MATLAB scripts^21^. ISPC was defined as

$${\mathrm{ISPC}}_{f}=\left|{n}^{-1}\sum_{t=1}^{n}{e}^{i\left(\theta xt-\left.\theta yt\right)\right.}\right|$$

where $n$ is the number of time points, and $\theta x$ and $\theta y$ are phase angles from electrodes *x* and *y* at frequency $f$ for trial $t$. $i$ is the imaginary operator.${n}^{-1}\sum_{t=1}^{n}(\cdot )$

denotes averaging across trials. ${e}^{i(\theta xt-\theta yt)}$ denotes a complex vector with magnitude 1 and angle $\theta x-\theta y$^21^. We mainly reported imISPC, which ignores zero phase-lag connectivity to compensate for the influence of volume conduction^22^. The equation used for ImISPC differs from that used for ISPC only in terms of the imaginary part of the spectral coherence. Although ISPC is sensitive to the number of trials used in the analyses, around 40 trials per condition should lead to a stable estimate for most frequency bands^21^. In the golf putting task, the trial counts for expert, amateur, and novice golfers were 58.75 ± 1.01, 59.38 ± 0.77, and 58.66 ± 1.35, respectively. To ameliorate the concerns that differences in the number of trial counts between the groups might confound the results, a one-way ANOVA was performed. The results showed no significant differences between the groups (*p* = 0.094). Thus, the concern of unequal number of trials may confound the findings could be lessened. The electrode pairings of interest were Cz–F3, Cz–F4, Cz–C3, Cz–C4, Cz–P3, Cz–P4, Cz–O1, Cz–O2, Cz–T7, and Cz–T8 for 8–13 Hz (Fig. 2). We used Fisher’s z transformation to ensure approximate normal distribution across subjects before performing the statistical analysis.

### Statistical analysis

Our experimental design was meant to delineate coherence between bilateral regions of the brain and a midline central electrode site (Cz) over the sensorimotor region. Given that differences in putting distance may influence the kinematics of putting, such as impact velocity and swing durations, we used the putting distance for each participant as the covariate to exclude this confounding factor. To determine the frequency specificity, the 4–7 Hz, 8–13 Hz, and 14–20 Hz imISPC estimates between the Cz electrode and the bilateral electrode sites in the 5 regions were subjected to a 3 (group: experts, amateurs, novices) × 2 (hemisphere: left, right) × 5 (region: frontal, central, parietal, occipital, temporal) 3-way MANCOVA. When the MANCOVA revealed significant effects, we further subjected the data to Student’s t-test. Furthermore, all post hoc tests were adjusted using a false discovery rate (FDR) to control for inflation of the Type I error due to multiple comparisons^46^. In addition, the effect sizes were calculated using the partial *η*^2^ statistic and Cohen’s d^47^. Before applying the FDR, we set α = 0.05.

We analyzed 8–13 Hz imISPC in the resting condition to determine whether it was specific to the golf putting task. Continuous EEG data were segmented into 2-s epochs to obtain the mean 8–13 Hz imISPC in the resting condition. The results were analyzed using a 3 (group: experts, amateurs, novices) × 2 (hemisphere: left, right) × 5 (region: frontal, central, parietal, occipital, temporal) ANOVA.

## Data Availability

The datasets generated and/or analyzed during the current study are available from the corresponding author on reasonable request.
